# Reliability of Average Daily Steps Measured Through a Consumer Smartwatch in Parkinson Disease Phenotypes, Stages, and Severities: Cross-Sectional Study

**DOI:** 10.2196/63153

**Published:** 2025-03-18

**Authors:** Edoardo Bianchini, Domiziana Rinaldi, Lanfranco De Carolis, Silvia Galli, Marika Alborghetti, Clint Hansen, Antonio Suppa, Marco Salvetti, Francesco Ernesto Pontieri, Nicolas Vuillerme

**Affiliations:** 1 Department of Neuroscience, Mental Health and Sensory Organs (NESMOS), Sapienza University of Rome, Rome, Italy; 2 AGEIS, Université Grenoble Alpes, Grenoble, France; 3 Department of Neurology, Kiel University, Kiel, Germany; 4 Department of Human Neurosciences, Sapienza University of Rome, Rome, Italy; 5 IRCCS Neuromed Institute, Pozzilli, Italy; 6 Institut Universitaire de France, Paris, France

**Keywords:** gait, Parkinson disease, phenotype, wearable sensors, smartwatch, step count, reliability, activity monitor, digital health technology, digital outcome measures, wearable, mHealth, motor, quality of life, fall, posture, mobile health

## Abstract

**Background:**

Average daily steps (avDS) could be a valuable indicator of real-world ambulation in people with Parkinson disease (PD), and previous studies have reported the validity and reliability of this measure. Nonetheless, no study has considered disease phenotype, stage, and severity when assessing the reliability of consumer wrist-worn devices to estimate daily step count in unsupervised, free-living conditions in PD.

**Objective:**

This study aims to assess and compare the reliability of a consumer wrist-worn smartwatch (Garmin Vivosmart 4) in counting avDS in people with PD in unsupervised, free-living conditions among disease phenotypes, stages, and severity groups.

**Methods:**

A total of 104 people with PD were monitored through Garmin Vivosmart 4 for 5 consecutive days. Total daily steps were recorded and avDS were calculated. Participants were dichotomized into tremor dominant (TD; n=39) or postural instability and gait disorder (PIGD; n=65), presence (n=57) or absence (n=47) of tremor, and mild (n=65) or moderate (n=39) disease severity. Based on the modified Hoehn and Yahr scale (mHY), participants were further dichotomized into earlier (mHY 1‐2; n=68) or intermediate (mHY 2.5‐3; n=36) disease stages. Intraclass correlation coefficient (ICC; 3,k), standard error of measurement (SEM), and minimal detectable change (MDC) were used to evaluate the reliability of avDS for each subgroup. The threshold for acceptability was set at an ICC ≥0.8 with a lower bound of 95% CI ≥0.75. The 2-tailed Student *t* tests for independent groups and analysis of 83.4% CI overlap were used to compare ICC between each group pair.

**Results:**

Reliability of avDS measured through Garmin Vivosmart 4 for 5 consecutive days in unsupervised, free-living conditions was acceptable in the overall population with an ICC of 0.89 (95% CI 0.85‐0.92), SEM below 10%, and an MDC of 1580 steps per day (27% of criterion). In all investigated subgroups, the reliability of avDS was also acceptable (ICC range 0.84‐0.94). However, ICCs were significantly lower in participants with tremor (*P*=.03), with mild severity (*P*=.04), and earlier stage (*P*=.003). Moreover, SEM was below 10% in participants with PIGD phenotype, without tremor, moderate disease severity, and intermediate disease stage, with an MDC ranging from 1148 to 1687 steps per day (18%‐25% of criterion). Conversely, in participants with TD phenotype, tremor, mild disease severity, and earlier disease stage, SEM was >10% of the criterion and MDC values ranged from 1401 to 2263 steps per day (30%‐33% of the criterion).

**Conclusions:**

In mild-to-moderate PD, avDS measured through a consumer smartwatch in unsupervised, free-living conditions for 5 consecutive days are reliable irrespective of disease phenotype, stage, and severity. However, in individuals with TD phenotype, tremor, mild disease severity, and earlier disease stages, reliability could be lower. These findings could facilitate a broader and informed implementation of avDS as an index of ambulatory activity in PD.

## Introduction

Walking is a fundamental motor ability, and it is pivotal for functional independence and social well-being [1]. Gait disturbances are common in people with Parkinson disease (PD), and include shuffling gait, shortened step length, altered automaticity, decreased arm swing, and freezing of gait [2]. These represent a particularly disabling group of symptoms, significantly hampering the quality of life of people with PD [3] and increasing the risk of falls [4].

Daily steps are an easy-to-collect and useful measure of ambulatory activity and mobility [5]. Although this parameter could not provide details regarding subtle gait features, several evidence linked a reduced daily step count to overall mortality risk [6-9], as well as to a range of health conditions, such as dementia [10] and oncological and cardiovascular diseases [11,12]. Previous studies have also reported a negative correlation between daily steps and disease severity in PD [13] and proposed a minimum daily step goal of 4200 to match with physical activity recommendations in the early stages of the disease [14].

Wearable devices, including smartwatches, could represent a useful option to estimate daily steps in an unobtrusive, ecological way [15]. Moreover, wearables are widely available on the market, easily used by the general population, and enable unobtrusive continuous long-term data collection [16]. However, since these devices are usually tested in healthy populations, knowledge of the validity and reliability of collected data is generally limited when applied to different groups of patients. Therefore, a growing body of literature focused on the validity and reliability of consumer wearable devices for step counting, with generally positive results [17-19].

In PD, motor and gait manifestations could render step detection and step count challenging and hence significantly diminish the validity and reliability of device algorithms [20-22]. Nevertheless, a prior study by Ginis et al [23] demonstrated a good criterion validity in estimating average daily steps (avDS) of 2 Fitbit devices (Fitbit Alta and Fitbit Inspire 3) in 28 people with PD in a real-life setting, compared to a research-grade device (Dynaport Movemonitor, McRoberts, NL).

Similarly, a prior study from our group involving 47 individuals with PD demonstrated a good criterion validity in step counting using a consumer smartwatch (Garmin Vivosmart 4), when worn on the side least affected by the disease and under well-controlled pharmacological conditions in a supervised, in-clinic setting [20].

Besides criterion validity, we recently demonstrated that optimal reliability of avDS recorded by Garmin Vivosmart 4 in real-life conditions could be achieved if the smartwatch is worn for a minimum of 4 days [24].

However, the clinical heterogeneity of PD when evaluating metrological characteristics of step-counting devices has been neglected so far. Indeed, the clinical presentation of PD is highly variable among individuals, thus, significant efforts have been made to identify distinct clusters and subtypes [25]. Several classifications have been proposed over the years from clinical-based [26-28] to more recent biomarker-based classifications [29-31]. In this regard, one of the most used classifications distinguishes PD with predominant features of tremor (ie, tremor dominant [TD]) or gait, posture, and balance issues (ie, postural instability and gait disorder [PIGD]) [26-28] based on subitems scores of the Unified Parkinson’s Disease Rating Scale (UPDRS) and its revision by the Movement Disorder Society (MDS-UPDRS) [32]. This classification could be relevant when measuring avDS in PD since tremors could increase the noise-to-signal ratio, making step detection more challenging [21]. Similarly, in PIGD individuals, the higher degree of gait alterations could alter the performance of step-detection algorithms [20,22].

Symptom severity and disease stage could also represent other relevant parameters to be considered when assessing the reliability of any wearable devices in counting avDS. Indeed, with disease progression, gait features increasingly deviate from normality [2,33], and tremor and bradykinesia could further alter the spatiotemporal and kinematic characteristics of walking, dampening, in turn, algorithm performance in step detection [20-22].

Nevertheless, no study to date considered disease phenotype, stage, and symptom severity when assessing the reliability of consumer wrist-worn devices for step counting in unsupervised, free-living conditions in mild-to-moderate people with PD. This study was hence specifically designed to address this issue. We hypothesized that reduced reliability might be observed in TD individuals and people with PD with more severe symptoms and in more advanced disease stages due to the aforementioned increased signal noise due to tremor, the higher degree of motor symptoms, and the more marked gait alterations. In addition, since no previous study specifically investigated the criterion validity of Garmin Vivosmart 4 in estimating avDS, we performed a proof-of-concept experiment in a subgroup of participants. Details of methods and results of this latter experiment are presented in Multimedia Appendix 1.

## Methods

### Population

Participants were consecutively screened and recruited during scheduled visits at the Movement Disorder Outpatient Service of the Sant’Andrea University Hospital (Rome, Italy) in the period between March 2023 and March 2024. Inclusion criteria were: (1) diagnosis of idiopathic PD according to the MDS criteria (Postuma et al [34]); (2) aged 18 years or older; (3) disease stage <4 according to the modified Hoehn and Yahr scale (mHY) [35]; (4) classification as TD or PIGD according to Stebbins et al [27]; and (5) stable medication in the 4 weeks before the experimental procedure. Exclusion criteria were: (1) cognitive impairment, defined by Montreal Cognitive Assessment [36] score <21; and (2) orthopedic, rheumatologic, or systemic conditions affecting mobility as judged by the assessor.

### Ethical Considerations

This cross-sectional study was performed in accordance with the ethical standards laid down in the 1964 Declaration of Helsinki and its later amendments. Approval was granted by the local Ethical Committee of Sapienza, University of Rome, Italy (0372/2022). Data collection and processing followed the current European regulations for data protection. All participants provided written informed consent before the beginning of measurements. All data were deidentified. Participants did not receive any form of compensation.

### Demographic and Clinical Data

Participants were evaluated during scheduled visits. Demographics and anthropometric measures (including age, sex, weight, height, and BMI) were collected. Disease duration and disease stage according to mHY and levodopa equivalent daily dose [37] were also collected. MDS-UPDRS [32] part III was used to assess motor symptoms severity.

Participants were divided into 4 subgroup pairs. Based on MDS-UPDRS part II and III scores, participants were classified into TD or PIGD disease subtypes according to Stebbins et al [27]. To evaluate the effect of tremor presence on device reliability, participants were also classified as those with and without tremor based on a score of ≥1 at item 2.10 of MDS-UPDRS part II. Concerning disease severity, participants were grouped into those with mild or moderate disease severity based on the MDS-UPDRS score as proposed by Martínez-Martín et al [38]. Similarly, participants were dichotomized in earlier (mHY 1‐2) or intermediate (mHY 2.5‐3) stages, based on mHY score.

### Experimental Procedure

Participants received the smartwatch Garmin Vivosmart 4 after the visit and were instructed to wear it at home for a minimum of 5 consecutive days, including at least 1 weekend day, on the wrist of the body side least affected by the disease [20]. No reminders or further instructions were provided to participants during registration. We chose a 5-day period since we demonstrated previously that a minimum of 4 days of monitoring is needed to reliably estimate daily step count in PD [24]. Each smartwatch was configured according to the producer’s recommendations and participants were asked to perform daily activities as usual. After 5 days, participants returned the smartwatch. The total daily number of steps for each day was recorded and avDS were calculated [24]. Compliance was assessed based on the participants’ dashboard data. The device recognizes that it is worn through heart rate and inertial motion unit signal. We considered all recording days with >80% wear time while awake and no interruption of device use greater than 3 hours to be valid. Details of methods of the proof-of-concept experiment regarding criterion validity of Garmin Vivosmart 4 are presented in Multimedia Appendix 2.

### Data and Statistical Analysis

The statistical analyses were performed using JASP (version 0.18.3.0; JASP Team), R (version 4.0.3; R Core Team), and RStudio (version 2023.12.0+369; R Foundation for Statistical Computing) for Windows. Descriptive statistics were calculated for the examined variables. The normality of distributions was assessed by histogram and residual plots inspection.

To evaluate the relative reliability for the 5-day monitoring period in the overall population and each subgroup, a 2-way intraclass correlation coefficient (ICC) with a fixed set of raters and averaged ratings was used (ICC (3,k), where k was the number of days of measurement), together with a custom R script. The following reference cut-off values for ICC interpretation were used [39]: excellent: >0.90; good: 0.75‐0.90; moderate: 0.50‐0.75; and poor: <0.50. The a priori threshold for acceptable ICC was set at a point estimate ≥0.80 with a lower bound of 95% CI ≥0.75 in accordance with a previous study from our group [24].

To compare ICCs between the 4 subgroup pairs, 2 methods were applied. First, standard errors and point estimates of ICCs were used to compute *t* statistics and perform 2-tailed independent groups Student *t* tests. Second, the CI overlap between each group pair was graphically and numerically assessed. Nonoverlapping CIs were considered indicators of significantly different ICCs [40]. Previous evidence underscored that a 95% CI overlap assessment could inflate the risk of type II error and suggested that an 83.4% CI could be a more powerful option [40-42]. Therefore, we adopted this method for CI overlap evaluation.

Standard error of measurement (SEM) and minimal detectable change (MDC) with a CI of 95% were used to compute the absolute reliability for the 5-day recordings in the overall population and each subgroup [43]. SEM and MDC were reported as absolute value and percentage of criterion measure (SEM% and MDC%, respectively). The criterion was the avDS count derived from the 5 days. For all analyses, the significance threshold was set at α<.05. All data were reported as mean (SD) or median (IQR) for numerical data and n (%) for categorical variables.

## Results

### Overview

A total of 104 people with PD were included in the study. All participants were monitored through Garmin Vivosmart 4 at home for a period of 5 consecutive days. No participants or days were excluded based on the prespecified compliance criteria. Participants took on average 5923 (SD 3014) daily steps, ranging from 357 to 12,620. Details of demographic, anthropometric, and clinical variables of the study population are shown in Table 1. The overlap between the subgroups is shown in Multimedia Appendix 1.

Overall, the results of the proof-of-concept experiment suggest that the smartwatch Garmin Vivosmart 4 is valid and decently accurate in estimating avDS in PD. Details are presented in the Multimedia Appendix 2.

**Table 1.** Demographic, anthropometric, and clinical characteristics of the study population.

	Overall (N=104)	TD^a^ (n=39)	PIGD^b^ (n=65)	Tremor (n=57)	No tremor (n=47)	Mild (n=65)	Moderate (n=39)	mHY^c^ 1‐2 (n=68)	mHY 2.5‐3 (n=36)
Age (years), mean (SD)	68.0 (8.4)	66.4 (9.0)	68.9 (8.0)	69.5 (7.9)	66.7 (8.8)	66.8 (8.7)	69.9 (7.7)	65.3 (7.8)	73.0 (7.3)
Height (cm), mean (SD)	171 (9.0)	173 (7.6)	170 (9.6)	170 (9.6)	172 (8.4)	173 (9.3)	168 (7.6)	173 (8.1)	168 (9.8)
Weight (kg), mean (SD)	75.7 (13.1)	76.7 (13.0)	75.1 (13.2)	75.0 (12.1)	76.3 (13.9)	77.2 (13.7)	73.2 (11.8)	77.7 (13.8)	71.9 (10.8)
BMI (kg/m^2^), mean (SD)	25.7 (3.4)	25.5 (3.6)	25.8 (3.4)	25.8 (3.0)	25.7 (3.8)	25.7 (3.5)	25.8 (3.4)	25.9 (3.7)	25.4 (3.0)
Sex (female), n (%)	34 (33)	11 (28)	23 (35)	19 (33)	15 (32)	22 (34)	12 (31)	22 (32)	12 (33)
Disease duration (years), mean (SD)	6.4 (4.4)	5.2 (4.4)	7.0 (4.4)	7.2 (3.9)	5.7 (4.8)	5.7 (4.5)	7.5 (4.2)	5.3 (4.3)	8.4 (4.1)
LEDD^d^ (mg), mean (SD)	553 (302)	418 (247)	634 (304)	623 (271)	495 (316)	489 (289)	659 (296)	453 (248)	741 (307)
mHY, median (IQR)	2 (2-2.5)	2 (1-2)	2 (2-2.5)	2 (2-2.5)	2 (2-2)	2 (2-2)	2.5 (2-3)	2 (2-2)	2.5 (2.5‐3)
MDS-UPDRS-III^e^, median (IQR)	27 (21‐32)	26 (18‐31)	29 (22‐33)	29 (22‐33)	26 (21‐32)	23 (19-29)	33 (29‐37)	23 (18‐29)	33 (29‐36)
avDS^f^, mean (SD)	5923 (3014)	6654 (2733)	5485 (3109)	4594 (2612)	7020 (2898)	6512 (2857)	4942 (3049)	6838 (2908)	4195 (2419)

a TD: tremor dominant.

b PIGD: postural instability and gait disorder.

c mHY: modified Hoehn and Yahr scale.

d LEDD: levodopa equivalent daily dose.

e MDS-UPDRS III: Movement Disorder Society Unified Parkinson’s Disease Rating Scale part III.

f avDS: average daily steps.

### Reliability of avDS in People With PD

#### Relative Reliability

AvDS collected during 5 consecutive days showed a level of relative reliability above the threshold of acceptability, as indicated by an ICC point estimate of ≥0.80 and a lower 95% CI limit of ≥0.75, in the overall population and all subgroups. Moreover, daily step count showed excellent reliability in PD in the intermediate disease stage. Details of ICC and CI limits are shown in Table 2.

**Table 2.** ICC^a^ (3,k) values with 95% CI for the overall population and each subgroup^b^.

	Overall (N=104)	TD^c^ (n=39)	PIGD^d^ (n=65)	Tremor (n=57)	No tremor (n=47)	Mild (n=65)	Moderate (n=39)	mHY^e^ 1‐2 (n=68)	mHY 2.5‐3 (n=36)
ICC (3,k)	0.888	0.854	0.899	0.838	0.914	0.856	0.919	0.839	0.939
Lower 95% CI	0.850	0.767	0.855	0.760	0.868	0.793	0.871	0.769	0.900
Upper 95% CI	0.919	0.916	0.933	0.896	0.947	0.905	0.953	0.892	0.966
Lower 83.4% CI	N/A^f^	0.797	0.869	0.786	0.883	0.814	0.887	0.792	0.914
Upper 83.4% CI	N/A	0.901	0.925	0.881	0.939	0.893	0.945	0.878	0.959

a ICC: intraclass correlation coefficient.

b For subgroups, 83.4% CI to assess intervals overlap are also reported.

c TD: tremor dominant.

d PIGD: postural instability and gait disorder.

e mHY: modified Hoehn and Yahr scale.

f Not applicable.

#### Absolute Reliability

AvDS showed an SEM below 10% in the overall population with an MDC of 1580 (26.7% of the criterion). AvDS also showed an SEM below 10% in the PIGD disease subtype, in participants without tremor, with a moderate disease severity, and in an intermediate disease stage with an MDC ranging from 1148 to 1687 steps per day (18% to 25% of criterion). Conversely, in the TD disease subtype, in participants with tremor, with mild disease severity and in an early disease stage, SEM was >10% of criterion and MDC values ranged from 1401 to 2263 steps per day (30% to 33% of the criterion). Details of SEM and MDC are shown in Table 3.

**Table 3.** Absolute and percentage values of SEM^a^ and MDC^b^ for the overall population and each subgroup.

	Overall (N=104)	TD^c^ (n=39)	PIGD^d^ (n=65)	Tremor (n=57)	No tremor (n=47)	Mild (n=65)	Moderate (n=39)	mHY^e^ 1‐2 (n=68)	mHY 2.5‐3 (n=36)
SEM	570	742	495	506	609	701	414	817	267
SEM%	9.6	11.1	9.0	11.0	8.7	10.8	8.4	11.9	6.4
MDC	1580	2056	1372	1401	1687	1944	1148	2263	741
MDC%	26.7	30.9	25.0	30.5	24.0	29.8	23.2	33.1	17.7

a SEM: standard error of measurement.

b MDC: minimal detectable change.

c TD: tremor dominant.

d PIGD: postural instability and gait disorder.

e mHY: modified Hoehn and Yahr scale.

### Reliability Comparison Between Subgroups

When comparing ICCs between subgroups pairs, 2-tailed Student *t* test for independent groups showed a significant difference between participants with and without tremor (*t*_102_=1.897; *P*=.03), between PD with mild and moderate disease severity (*t*_102_=1.765; *P*=.04), and between individuals in early and intermediate disease stage (*t*_102_=2.817; *P*=.003). Conversely, no significant difference was found between TD and PIGD participants (*t*_102_=1.048; *P*=.15).

**Figure 1.**
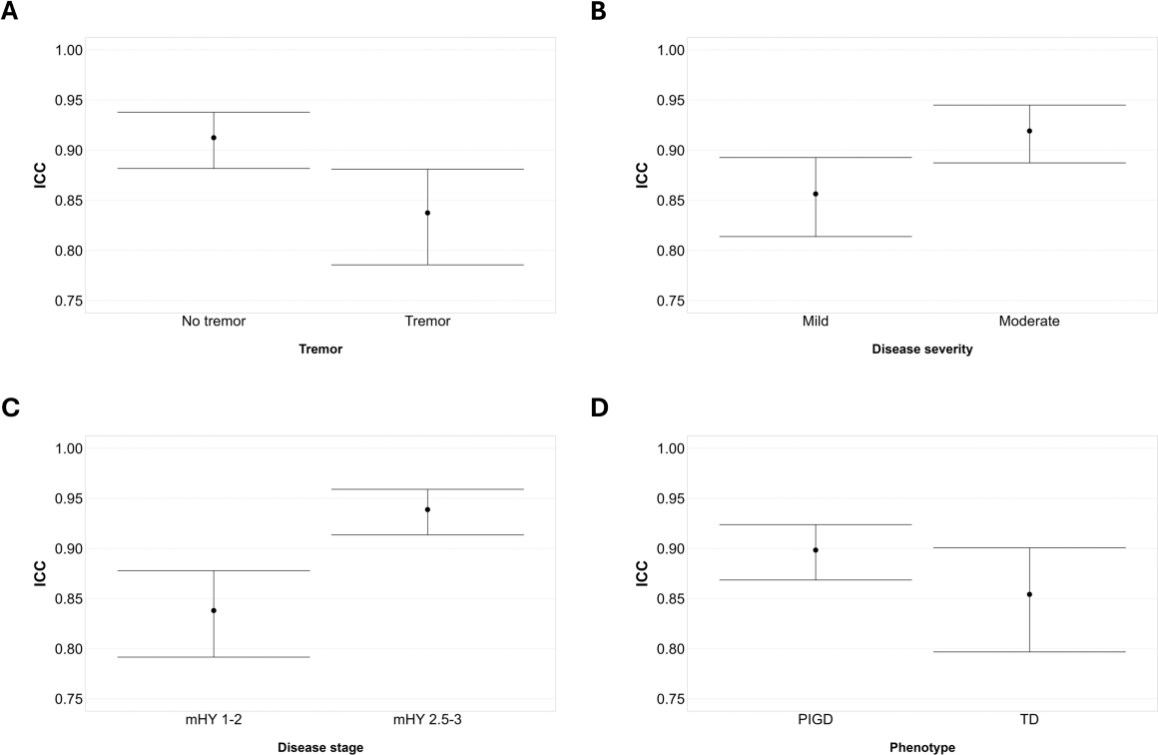
ICC and 83.4% CI comparison for each subgroup pair. (A) Participants with reported presence or absence of tremor. (B) Participants with mild and moderate disease severity as indicated by MDS-UPDRS. (C) Participants with earlier (mHY 1‐2) and intermediate (mHY 2.5‐3) disease stage. (D) PIGD and TD phenotypes. ICC (3,k) point estimate is indicated by the black dot, CI limits are represented by the vertical bars. ICC: intraclass correlation coefficient; MDS-UPDRS: International Movement Disorders Society Unified Parkinson’s Disease Rating Scale; mHY: modified Hoehn and Yahr stage; PIGD: postural instability and gait disorder; TD: tremor dominant.

The analysis of 83.4% CI showed no overlap between interval limits between participants with and without tremor (Figure 1A), in early and intermediate disease stage (Figure 1C), and only a negligible overlap between individuals with mild and moderate disease severity (Figure 1B). Conversely, a degree of overlap of the two 83.4% CIs was observed between TD and PIGD participants (Figure 1D). Details of 83.4% CI limits are shown in Table 2.

## Discussion

### Principal Findings

This cross-sectional study aimed at assessing and comparing the reliability of a consumer wrist-worn smartwatch (Garmin Vivosmart 4) in counting avDS in PD in unsupervised, free-living conditions for 5 consecutive days between disease phenotypes, stages, and severity groups.

Overall, our results showed, for the first time, that avDS were acceptably reliable in mild-to-moderate PD irrespective of disease severity, stage, or phenotype. Nevertheless, our results further indicated lower reliability in people with PD with TD phenotype, tremor, lower disease severity, and earlier stage.

### Reliability of avDS in the Overall PD Population

We found that the relative reliability of avDS, measured in unsupervised, free-living conditions, by Garmin Vivosmart 4 for 5 consecutive days was within the a priori criteria for acceptability in the overall PD population. We identified only 2 studies investigating the reliability of wearable devices in measuring avDS in people with PD [24,44]. Paul et al [44] reported that 2 consecutive days of monitoring were sufficient to obtain an ICC of >0.9, using a research-grade, ankle-mounted, step counter (Step Activity Monitor) in 92 people with PD. One study from our group investigated the reliability of a wrist-worn consumer device in measuring avDS in PD before. In that study, we found an ICC (3,k) of 0.88 (0.82‐0.93) for 4 days of monitoring in 56 mild-to-moderate people with PD using Garmin Vivosmart 4 [24]. The present results are consistent with our previous study, but also with other studies investigating the reliability of avDS through wrist-worn wearables in healthy older people [44-47], and in people with various neurological conditions, such as multiple sclerosis [48] and stroke [49]. Regarding absolute reliability, we found that SEM% was below 10% (9.6%) with an MDC of 1580 steps per day (27% of criterion). Only 1 study, from our group, investigated SEM and MDC for avDS in PD [24]. The present results are consistent with our previous study in which we reported an SEM% of 9.2% and an MDC of 1495 steps per day (or 26% of criterion) [24]. Taken together, these results confirm the reliability of avDS measured through Garmin Vivosmart 4 in unsupervised, free-living conditions in mild-to-moderate PD for 5 consecutive days.

### Reliability of avDS in People With PD Subgroups

To our knowledge, this is the first study to investigate and compare the reliability of a consumer smartwatch in measuring avDS in different subgroups of PD based on disease phenotype, severity, and stage. We found that in all investigated subgroups (ie, TD vs PIGD, mild vs moderate, tremor vs nontremor, and earlier vs intermediate), ICC values were within the a priori criteria for acceptability (ICC range 0.84‐0.94). However, a significantly lower ICC was observed in people with PD with tremor, mild disease severity, and earlier disease stage. Moreover, SEM% was below 10% in participants with PIGD phenotype, moderate disease severity, intermediate disease stage, and without tremor, with an MDC ranging from 1148 to 1687 steps per day (18%‐25% of criterion). Conversely, in individuals with TD phenotype, tremor, mild disease severity, and earlier disease stage, SEM was >10% of criterion and MDC values ranged from 1401 to 2263 steps per day (30%‐33% of the criterion).

MDC, defined as the minimal change that falls outside the measurement error of an instrument, is extremely relevant in study design since it allows to calculate the sample size of studies aiming to assess the effectiveness of interventions [50]. MDC could be also crucial to define the appropriateness and feasibility of using a determinate device to measure a given construct. A prior work from Handlery et al [14] reported an increase in 1250 steps per day following a high-intensity physical activity intervention in PD measured through a research-grade wrist-worn device (Actigraph GT3X). In this study, we found that in individuals with TD phenotype, mild disease severity, and earlier disease stage, MDC was ~2000 steps per day. Although a direct comparison with the metrics reported in the work from Handlery et al [14] could not be performed due to the different devices, we could hypothesize that only large modifications in avDS could be reliably measured through Garmin Vivosmart 4 in the aforementioned PD subgroups. To this end, future studies will be needed to define the minimal clinically important difference for avDS measured through consumer wrist-worn devices and to assess the attainability of avDS modifications sufficiently large to be reliably detected by these devices.

The reduced reliability in participants with tremor is in line with our hypothesis that tremor could reduce the performance of the step-detection algorithm. In fact, tremor could increase the noise-to-signal ratio in the accelerometer signal, and in turn, render step detection more challenging [21]. Indeed, a previous study highlighted that tremor and dyskinesia together contributed to more than 19% of the variation in daily step counts when comparing measurements from waist-worn and wrist-worn devices in 46 people with PD with similar characteristics to those included in this study [21]. In this regard, our study further supports the assumption that tremor could reduce the step-detection performance of wrist-worn devices in PD.

On the other hand, our hypothesis that a reduced reliability might be observed in participants with higher symptom severity and more advanced stages was not supported by our results. Indeed, the reduced reliability of avDS observed in individuals with mild disease severity and earlier disease stage is somehow counterintuitive. In fact, previous evidence highlighted that step count was less accurate in people walking at slower gait speed and with shorter step length in several neurological and musculoskeletal conditions, including PD [20,22,51-54]. Reduced step length and slower walking speed are typical features of Parkinsonian gait, with a higher prevalence along the disease course [2,33]. Moreover, another typical characteristic of walking in PD is the reduced automaticity that leads to a more discontinuous and irregular gait pattern that can further reduce device accuracy in step detection [2,22].

Despite these considerations, our results showed that avDS estimation was more reliable in people with PD with moderate disease severity and intermediate disease stage, compared with individuals with mild disease severity and earlier disease stage. In this regard, it must be considered that reliability is a measure of consistency and reproducibility of measurement and not a measure of accuracy [55]. Therefore, a reduced accuracy could not directly translate into reduced reliability. We could hypothesize that in more advanced stages of PD, the variability of clinical presentation could be lower. In an earlier stage, indeed, symptom heterogeneity, both in terms of motor and nonmotor features, could be extremely high [56-58]. However, this variability could decrease with disease progression since motor symptoms tend to consistently worsen along the disease course and motor features such as gait and balance impairment become increasingly prevalent [59,60]. In addition, the phenotype is dynamic along the disease course and some researchers have proposed that the classification into PIGD/TD evolves over time [61,62]. One study, indeed, reported that over a period of 8 years, approximately 70% of TD individuals transitioned to PIGD, whereas only 4% of PIGD individuals transitioned to TD [61]. Another study reported that 45% of TD participants at baseline had a subtype shift along a 2-year follow-up while 85% of PIGD participants remained as PIGD [62]. This is mirrored in our study cohort, where 32 out of 65 (49%) individuals with mild disease severity were classified as TD, whereas only 7 out of 39 (18%) were in the moderate group. Similarly, 33 out of 68 (49%) participants with earlier disease stage were classified as TD, whereas only 6 out of 36 (17%) were in the intermediate group. Therefore, a regression toward a more uniform motor impairment along the disease course might be considered. Moreover, since we found that tremor could be a relevant factor in reducing avDS reliability, the different prevalence of TD phenotype could also contribute to explaining our results. However, it must be underlined that no study, to our knowledge, systematically compared the heterogeneity of PD features across early, intermediate, and advanced disease stages. Therefore, our hypothesis should be taken with caution and future studies are needed to confirm it.

In conclusion, our findings highlight that, although avDS were reliable across the examined subgroups, clinicians and researchers should consider disease phenotype, stage, and severity when implementing wrist-worn wearables and interpreting mobility data collected through these devices in PD.

### Limitations

We acknowledge that this study has some limitations. First, the participants included in our study displayed relatively preserved cognitive functions, due to our exclusion of participants with a Montreal Cognitive Assessment of <21. Additionally, those with more advanced disease stages or requiring walking aids were not included. This potentially limits the generalizability of our findings. However, it should be considered that the sample in our study can be seen as representative of the typical target for interventions using consumer-grade wearable technology. Moreover, including individuals with a disease stage >3, using walking aids, or with more severe cognitive impairments poses significant challenges in the utilization of consumer technology and is beyond our scope. Nevertheless, future research incorporating PD with lower functional scores, higher disease severity, and more impactful cognitive impairments would be valuable. Furthermore, we used only PD subtyping based on clinical features, yet other classification methods and clustering techniques have been proposed incorporating instrumental and biological data. Future studies are warranted to investigate the reliability of consumer smartwatches in PD subgroups defined using multimodal biomarkers. Finally, we did not account for antiparkinsonian treatment to control tremor. All included participants were under dopaminergic medical treatment and stable medical treatment in the 4 weeks before data collection, and we did not enroll drug-naïve individuals. In addition, we did not account for different drug classes since it would have required a much higher sample size to cover all the possible combinations. Future studies might investigate the metrological features of commercial smartwatches in drug-naïve PD to avoid the influencing factor of dopaminergic treatment. However, since almost all patients with a diagnosis of PD are medically treated, we considered it relevant to assess the measurement properties of commercial devices in a setting more representative of the real-world experience.

### Conclusions

In mild-to-moderate PD, avDS measured through a consumer smartwatch in unsupervised, free-living conditions for 5 consecutive days are reliable irrespective of disease phenotype, stage, and severity. Researchers and clinicians who want to implement these instruments should consider that in individuals with TD phenotype, tremor, mild disease severity, and earlier disease stage, reliability could be lower and MDC could be higher. Future studies are needed to define the minimal clinically important difference for avDS measured through consumer wrist-worn devices and to assess the attainability of avDS modifications sufficiently large to be reliably detected by wrist-worn consumer devices. Taken together, we believe that these results could facilitate a broader implementation and an informed application of avDS as an index of ambulatory activity in PD and could be highly relevant to developing monitoring, preventive, educational, and rehabilitation strategies for PD.

## Supplementary material

10.2196/63153 Multimedia Appendix 1 Overlap between the groups of participants with and without self-reported tremor and TD or PIGD phenotypes as well as between the groups with mild or moderate disease severity and early or intermediate disease stage. mHY: modified Hoehn and Yahr scale; PIGD: postural instability and gait disorder; TD: tremor dominant.

10.2196/63153 Multimedia Appendix 2 Proof-of-concept experiment to test criterion validity of Garmin Vivosmart 4 in estimating average daily steps in free-living conditions in people with Parkinson disease.

## Abbreviations

avDS: average daily steps
ICC: intraclass correlation coefficient
MDC: minimal detectable change
MDS-UPDRS: International Movement Disorders Society Unified Parkinson’s Disease Rating Scale
mHY: modified Hoehn and Yahr scale
PD: Parkinson disease
PIGD: postural instability and gait disorder
SEM: Standard error of measurement
TD: tremor dominant
