# Supplementary material for: Reliability of Average Daily Steps Measured Through a Consumer Smartwatch in Parkinson Disease Phenotypes, Stages, and Severities: Cross-Sectional Study
Source: JMIR Form Res. 2025 Mar 18;9:e63153. doi: 10.2196/63153 (PMC11936306; doi:10.2196/63153)
Supplement: Multimedia Appendix 1 [file formative-v9-e63153-s001.docx]

**Table S1.** Overlap between the groups of patients with and without selfreported tremor and TD/PIGD phenotypes. PIGD: postural instability and gait disorder; TD: tremor dominant.

|  | TD | PIGD | Tremor | No tremor |
| --- | --- | --- | --- | --- |
| TD | - | - | 36/57 (63%) | 3/47 (6%) |
| PIGD | - | - | 21/57 (37%) | 44/47 (93%) |
| Tremor | 36/39 (92%) | 44/65 (68%) | - | - |
| No tremor | 3/39 (8%) | 21/65 (32%) | - | - |
|  | 39 | 65 | 57 | 47 |

**Table S2.** Overlap between the groups of patients with mild/moderate disease severity and early/intermediate disease stage. mHY: modified Hoehn and Yahr scale.

|  | Mild | Moderate | mHY 1-2 | mHY 2.5-3 |
| --- | --- | --- | --- | --- |
| Mild | - | - | 52/68 (76%) | 13/36 (36%) |
| Moderate | - | - | 16/68 (24%) | 23/36 (64%) |
| mHY 1-2 | 52/68 (80%) | 16/39 (41%) | - | - |
| mHY 2.5-3 | 13/68 (20%) | 23/39 (59%) | - | - |
|  | 65 | 39 | 68 | 36 |
