# Supplementary material for: Reliability of Average Daily Steps Measured Through a Consumer Smartwatch in Parkinson Disease Phenotypes, Stages, and Severities: Cross-Sectional Study
Source: JMIR Form Res. 2025 Mar 18;9:e63153. doi: 10.2196/63153 (PMC11936306; doi:10.2196/63153)
Supplement: Multimedia Appendix 2 [file formative-v9-e63153-s002.docx]

Since no previous study specifically investigated the criterion validity of the smartwatch Garmin Vivosmart 4 in estimating average daily steps (avDS), we performed this proof-of-concept experiment in a small subgroup people with Parkinson’s disease (PwPD).

## Methods

Details regarding population, ethics and collected clinical measures are presented in the main manuscript.

### Experimental procedure

Participants were provided with a smartwatch Garmin Vivosmart 4 and a lower-back mounted research-grade inertial sensor for mobility analysis (McRoberts MoveMonitor, McRoberts B.V., Den Haag, The Netherlands) to wear concurrently for 7 consecutive days in their home environment. They were instructed to wear both the smartwatch on the wrist of the body side least affected by the disease and MoveMonitor all time during day and night and remove them only when involved in water activities (e.g., bathing, showering, swimming, etc.). As far as Garmin Vivosmart 4 is concerned, data extraction and compliance assessment are described in the main section of the manuscript. For MoveMonitor, the number of steps taken each day were extracted from device report and the avDS were calculated. Compliance was assessed through the “no wear time” provided by device report. We considered all recording days with ≥80% wear time while awake for both devices to be valid.

### Data and statistical analysis

The statistical analyses were performed using JASP v0.18.3.0 (JASP Team, University of Amsterdam, The Nederlands). Statistical analyses were performed using JASP v0.18.3 (JASP Team, University of Amsterdam, Amsterdam, The Netherlands).

To assess the agreement between the number of steps counted by Garmin Vivosmart 4 and MoveMonitor (gold standard), the intraclass correlation coefficient (2,1) (ICC_2,1_) was used and the point estimate and lower and upper 95% confidence interval reported. The following reference cut-off values for the ICC interpretation were used [1]: Excellent: >0.90; Good: 0.75–0.90; Moderate: 0.50–0.75; Poor: <0.50. From the number of steps counted by Garmin Vivosmart 4 and MoveMonitor, the mean percentage error (MPE) and the mean absolute percentage error (MAPE) were calculated for avDS as follows:

$$MPE=\frac{\left( steps counted by MoveMonitor \right) - (steps counted by smartwatch)}{(steps counted MoveMonitor)}\times100$$

$$MAPE=\frac{\left| \left( steps counted MoveMonitor \right) - (steps counted by smartwatch) \right|}{(steps counted MoveMonitor)}\times100$$

The MPE was used to evaluate the direction-of-error of the smartwatch Garmin Vivosmart 4 compared with MoveMonitor. A negative value indicated an overestimate of counting and vice versa. The MAPE was used to assess the magnitude-of-error of the smartwatch Garmin Vivosmart 4 in step counting compared with MoveMonitor.

## Results

Data from a subgroup of 11 PwPD [Age: 66.4 ± 7.0 years; Females: 4 (36%); Disease duration: 7.9 ± 3.9 years; Hoehn & Yahr: 2 (2-2.5)] were analyzed for a total of 64 valid days of recording for both devices. The median recording time was 6 days (range 5-7).

The smartwatch Garmin Vivosmart 4 demonstrated an excellent agreement with MoveMonitor for avDS [ICC_2,1_ 0.984 = (0.946 – 0.996)]. MPE was -1.3% (CI95%: -11.0 – 8.4) and MAPE was 10.2% (CI95%: 3.6 – 16.8) for avDS.

## Discussion

In this proof-of-concept experiment, the smartwatch Garmin Vivosmart 4 demonstrated an excellent agreement with MoveMonitor research-grade inertial sensor. It is important to mention that MoveMonitor was extensively validated both technically and clinically [2,3] and was used for several large multicentric studies in PwPD to monitor gait in free-living, real-world conditions as part of international consortia (e.g., Mobilize-D). Moreover, MoveMonitor was used in a previous study to demonstrate the criterion validity of Fitbit Inspire in measuring avDS [4]. Our result is in line with those reported in this previous study which showed a good-to-excellent ICC (>0.83) between the smartwatch and the research-grade device. Moreover, although preliminary, the present results confirm the findings from a previous study from our group showing a good agreement between steps measured through Garmin Vivosmart 4 and manual counting in a supervised setting [5].

Regarding MPE and MAPE, our preliminary findings point towards a slight overestimation of avDS by the smartwatch. This result is in line with our preliminary findings in supervised setting. Regarding MAPE Garmin Vivosmart 4 showed an error around 10% in estimating avDS. To the best of our knowledge, no previous study reported this aspect in PwPD. However, our results are in line with or lower than previous studies on healthy individuals [6]. Moreover, it is worth noting that the MAPE is within the threshold of acceptability as indicated by the cut-off value of 15% recommended for devices intended to be used by the general population [7].

In conclusion, our proof-of-concept experiment suggests that the smartwatch Garmin Vivosmart 4 is valid and decently accurate in estimating avDS in PwPD. It must be stressed, however, that this is a proof-of-concept experiment, and the results hereby presented were included since no previous study are available to support the criterion validity of the used smartwatch. Therefore, these findings are not sufficient to conclusively demonstrate the accuracy and validity of the smartwatch Garmin Vivosmart 4. Further studies are hence warranted to fully address this issue.

## References

1. Koo TK, Li MY. A Guideline of Selecting and Reporting Intraclass Correlation Coefficients for Reliability Research. J Chiropr Med 2016 Jun;15(2):155–163. PMID:27330520

2. Micó-Amigo ME, Bonci T, Paraschiv-Ionescu A, Ullrich M, Kirk C, Soltani A, Küderle A, Gazit E, Salis F, Alcock L, Aminian K, Becker C, Bertuletti S, Brown P, Buckley E, Cantu A, Carsin A-E, Caruso M, Caulfield B, Cereatti A, Chiari L, D’Ascanio I, Eskofier B, Fernstad S, Froehlich M, Garcia-Aymerich J, Hansen C, Hausdorff JM, Hiden H, Hume E, Keogh A, Kluge F, Koch S, Maetzler W, Megaritis D, Mueller A, Niessen M, Palmerini L, Schwickert L, Scott K, Sharrack B, Sillén H, Singleton D, Vereijken B, Vogiatzis I, Yarnall AJ, Rochester L, Mazzà C, Del Din S, Mobilise-D consortium. Assessing real-world gait with digital technology? Validation, insights and recommendations from the Mobilise-D consortium. J Neuroeng Rehabil 2023 Jun 14;20(1):78. PMID:37316858

3. Mazzà C, Alcock L, Aminian K, Becker C, Bertuletti S, Bonci T, Brown P, Brozgol M, Buckley E, Carsin A-E, Caruso M, Caulfield B, Cereatti A, Chiari L, Chynkiamis N, Ciravegna F, Del Din S, Eskofier B, Evers J, Garcia Aymerich J, Gazit E, Hansen C, Hausdorff JM, Helbostad JL, Hiden H, Hume E, Paraschiv-Ionescu A, Ireson N, Keogh A, Kirk C, Kluge F, Koch S, Küderle A, Lanfranchi V, Maetzler W, Micó-Amigo ME, Mueller A, Neatrour I, Niessen M, Palmerini L, Pluimgraaff L, Reggi L, Salis F, Schwickert L, Scott K, Sharrack B, Sillen H, Singleton D, Soltani A, Taraldsen K, Ullrich M, Van Gelder L, Vereijken B, Vogiatzis I, Warmerdam E, Yarnall A, Rochester L. Technical validation of real-world monitoring of gait: a multicentric observational study. BMJ Open 2021 Dec 2;11(12):e050785. PMID:34857567

4. Ginis P, Goris M, De Groef A, Blondeel A, Gilat M, Demeyer H, Troosters T, Nieuwboer A. Validation of Commercial Activity Trackers in Everyday Life of People with Parkinson’s Disease. Sensors (Basel) 2023 Apr 21;23(8):4156. PMID:37112496

5. Bianchini E, Caliò B, Alborghetti M, Rinaldi D, Hansen C, Vuillerme N, Maetzler W, Pontieri FE. Step-Counting Accuracy of a Commercial Smartwatch in Mild-to-Moderate PD Patients and Effect of Spatiotemporal Gait Parameters, Laterality of Symptoms, Pharmacological State, and Clinical Variables. Sensors (Basel) 2022 Dec 25;23(1):214. PMID:36616812

6. Germini F, Noronha N, Borg Debono V, Abraham Philip B, Pete D, Navarro T, Keepanasseril A, Parpia S, de Wit K, Iorio A. Accuracy and Acceptability of Wrist-Wearable Activity-Tracking Devices: Systematic Review of the Literature. J Med Internet Res 2022 Jan 21;24(1):e30791. PMID:35060915

7. Johnston W, Judice PB, Molina García P, Mühlen JM, Lykke Skovgaard E, Stang J, Schumann M, Cheng S, Bloch W, Brønd JC, Ekelund U, Grøntved A, Caulfield B, Ortega FB, Sardinha LB. Recommendations for determining the validity of consumer wearable and smartphone step count: expert statement and checklist of the INTERLIVE network. Br J Sports Med 2021 Jul;55(14):780–793. PMID:33361276
